# Cornstarch is less allergenic than corn flour in dogs and cats previously sensitized to corn

**DOI:** 10.1186/s12917-018-1538-5

**Published:** 2018-06-27

**Authors:** Thierry Olivry, Jennifer Bexley

**Affiliations:** 1 Department of Clinical Sciences, College of Veterinary Medicine, North Carolina State University, 1060 Willliam Moore Drive, Raleigh, NC 27606 USA; 2 Comparative Medicine Institute, College of Veterinary Medicine, North Carolina State University, Raleigh, NC USA; 3 Avacta Animal Health, Wetherby, Leeds UK

**Keywords:** Allergen, Allergy, Canine, Diet, Food, Feline, Corn, IgE, Maize, Starch

## Abstract

**Background:**

Corn appears to be an uncommon food source of allergens in dogs and cats. There is limited information on the nature of the corn allergens in dogs and cats and their presence in the various foodstuffs used in commercial pet foods. The aim of this study was to determine if serum IgE from corn-sensitized dogs and cats recognized proteins in corn flour and cornstarch, which are common sources of carbohydrates in pet foods.

**Results:**

We selected archived sera from allergy-suspected dogs (40) and cats (40) with either undetectable, low, medium or high serum levels of corn-specific IgE. These sera were tested then by ELISA on plates coated with extracts made from corn kernels, corn flour, cornstarch and the starch used in the commercially-available extensively-hydrolyzed pet food Anallergenic (Royal Canin). Immunoblotting was then performed on the same extracts with some of the sera from moderate-to-high corn-sensitized dogs and cats. Using ELISA, it is mostly the dogs and cats with moderate and high corn-specific IgE levels that also had IgE identifying allergens in the flour (dogs: 20/30 sera, 67% - cats: 20/29, 69%). In contrast, none of the tested sera had measurable IgE against proteins isolated from the cornstarch. Immunoblotting confirmed the existence of numerous major corn allergens in the corn kernel extract, fewer in that of the corn flour, while such allergens were not detectable using this technique in the two cornstarch extracts.

**Conclusions:**

In this study, ELISA and immunoblotting results suggest that IgE from corn-sensitized dogs are less likely to recognize allergens in cornstarch than in kernel and flour extracts. As corn is not a common allergen source in dogs and cats, and as its starch seems to be less allergenic than its flour, pet foods containing cornstarch as a carbohydrate source are preferable for dogs and cats suspected of suffering from corn allergy.

**Electronic supplementary material:**

The online version of this article (10.1186/s12917-018-1538-5) contains supplementary material, which is available to authorized users.

## Background

Cutaneous adverse food reactions (CAFR) represent one of the most common diagnoses given to dogs and cats with pruritus or allergic skin diseases [1]. To echo the terminology used in human medicine [2], the term CAFR was first coined for animals with skin manifestations of food reactions that included both food allergies (that is, adverse health effects arising from a specific immune response occurring reproducibly on exposure to a given food) and food intolerances (e.g., reactions occurring following non-immune mechanisms). While we could not identify any well-documented case of non-immunologic food intolerance in dogs and cats in the veterinary literature, there are numerous studies suggesting an immunologic foundation to canine and feline CAFRs. Indeed, there is documentation of either food-specific IgE hypersensitivity and/or food-activated T cells in such patients (reviewed in [3]). That most dogs and cats with CAFR likely suffer from bona fide food allergies rather than food intolerances opens new avenues for a better diagnosis and treatment. For example, characterizing allergenic food sources and the main culprit allergens would likely lead to more accurate restriction-provocation trials, IgE serological and cell-mediated diagnostic assays. Furthermore, one could then also envision the possible development of oral allergen immunotherapy and the design of hypoallergenic pet foods for the long-term management of food allergic dogs and cats.

A recent critically appraised topic concluded that the most common allergenic food sources in dogs and cats are derived from mammalian, poultry, and fish meats, while grains are uncommon culprits [4]. Among the latter, corn was found to be an offending allergen in less than 5% of dogs and cats with food allergies [4].

Recent proteomic studies markedly expanded the list of possible IgE-targeted corn allergenic proteins for humans [5–7]. An important observation of the most recent studies was that corn allergens appear to vary between plant cultivars as well as between allergic human individuals [6, 7]. The major corn allergen Zea m 14, recognized by nearly 90% of patients, is a 9 kDa lipid transfer protein present in corn kernels [8].

To date, there is only limited information available on the identity of corn allergens in dogs, while none has been reported yet in cats. Cornstarch is frequently added to commercial pet foods, including extensively-hydrolyzed ones. Whilst there is no clinical evidence to suggest its inclusion in such diets would be problematic for animals sensitized to corn, in a recent study evaluating the proteins present in three commercial hydrolysate-containing pet foods, granule-bound starch synthase 1 (GBSS-I, an NDP-glucose-starch glucosyltransferase) was the only corn protein identified as an allergen by serum IgE from a small fraction of dogs with suspected nonseasonal allergy [9]; of importance is that GBSS-I is present in cornstarch.

The objectives of this study were to determine if IgE from corn-sensitized dogs and cats recognized proteins present in corn flour and starch. We will show herein that, presumably due to its very low protein content and limited proteome, cornstarch is hypoallergenic compared to its flour.

## Methods

### Animal sera

We used canine and feline leftover sera submitted to Avacta Animal Health (Wetherby, Leeds, UK) for determination of food allergen-specific IgE serological testing.

For these studies, sera from dogs and cats exhibiting signs suggestive of an allergic dermatitis were chosen based on their level of corn-specific IgE, but information whether or not these patients suffered from corn-induced clinical allergies was not available. All sera had been kept at − 80 °C until assayed.

We selected canine and feline sera according to their original levels of serum corn-specific IgE (IgE reactivities expressed as optical density, OD, values at 405 nm, OD_405_ uncorrected for background) in the Sensitest commercially available ELISA (Avacta). The initial screening had been made with a standard corn extract (SCE; F102, Greer laboratories, Lenoir, North Carolina, USA).

For ELISA studies, we selected 40 canine sera divided as follows:

- D1-LCoR: ten dogs with low corn IgE reactivity and a previous OD_405_ (uncorrected) between 0.200 and 0.500 in the Sensitest assay.
- D1-MCoR: ten dogs with moderate IgE corn reactivity (OD_405_ between 0.501 and 1.000)
- D1-HCoR: ten dogs with high corn IgE reactivity (OD_405_ > 1.000)
- D2-NCoR: ten dogs with undetectable levels of IgE to corn (i.e. “non-corn reactive dogs”; OD_405_ < 0.200).

Similarly, we retained 40 feline sera using identical OD_405_ brackets as for dogs; The categories were as follows:

- C1-LCoR: nine cats with low corn IgE reactivity
- C1-MCoR: ten cats with moderate corn IgE reactivity
- C1-HCoR: ten cats with high corn IgE reactivity
- C2-NCoR: 11 cats with undetectable corn IgE

For immunoblotting, we selected ten dog sera among those with moderate-to-high corn IgE reactivity, as defined above. A negative pool included five sera with undetectable levels of corn-specific IgE.

Due to the rarity of corn-sensitized cats, we only had sufficient serum from nine cats with low-to-high corn-specific IgE to be used for this technique; a negative pool was selected as for dogs.

### Corn extracts

Were tested the standard corn (kernel) extract (SCE) from Greer laboratories (F102), and others made from regular corn flour (RCF), regular cornstarch (RCS) and the purified cornstarch used in Royal Canin Anallergenic (Anallergenic cornstarch, ACS); the last three food sources were provided by Royal Canin (Aimargues, France).

All extracts but the SCE were prepared as follows: allergens were extracted with PBS, pH 7.4, for 4 h at 2–8 °C (material/PBS ratio of 1:10 *w*/*v*) before being centrifuged at 15,000 g for 20 min. The supernatant was retained at 2–8 °C, and the pellet was further extracted with PBS overnight at 2–8 °C. Following the second centrifugation, the supernatant was collected again and combined with the previous one. The protein concentration of the extract was determined by measurement at 280 nm (Trinean DropSense 96, Gentbrugge, Belgium).

### ELISAs

Food allergen-specific IgE serum levels were determined by ELISA as described previously [10]. Herein, and as before, we also used 250 ng/protein of each of the four extracts per well and 50 μL of dog or cat sera diluted at 1:10.

For standardization of the canine and feline ELISAs, serial three-fold dilutions of pools of dog or cat reference sera with high levels of anti-corn IgE were included on each plate. Undiluted, these two standard pools were assigned a value of 500 arbitrary units (AU).

Positive and negative serum controls, with moderate and negligible levels of corn specific IgE, respectively, were also diluted at 1:10 and included allergen-coated and uncoated (no-coat control) plates.

### Electrophoresis and immunoblotting

The tested extracts were the same as those used for ELISA (SCE, RCF, RCS and ACS); they were heated at 70 °C for 10 min in the presence of a reducing agent (Expedeon, Cambridge, UK) before sodium dodecyl sulphate polyacrylamide gel electrophoresis (SDS-PAGE) electrophoresis. Approximately 5 μg of protein was deposited in each lane and, except for minor changes in reagent suppliers, SDS-PAGE and immunoblotting were otherwise performed similarly to our recent report [10].

## Results

### Extract preparation

The protein yields for the RCF, RCS, and ACS averaged from three separate measurements, were 1.63, 0.13 and 0.27 mg/ml, respectively. These concentrations were sufficient for coating plates with a 5 μg/mL solution.

### ELISA validation

The canine ELISA standard curve had an accuracy of between 100 and 106% at concentrations varying between 3.7 and 100 AU. The inter-assay coefficient of variation of the positive control was 4%. We established the positive threshold of detection at 10.0 AU as the mean OD_405_ plus three standard deviations of 95 canine sera without detectable anti-corn IgE.

For the feline ELISA, the standard curve was 92–113% accurate over the same concentration range as above. The inter-assay coefficient of variation of the positive control was 1.1%. The positive threshold, determined as for the canine ELISA, was calculated to be 12.7 AU.

### Canine ELISAs

**Fig. 1:**
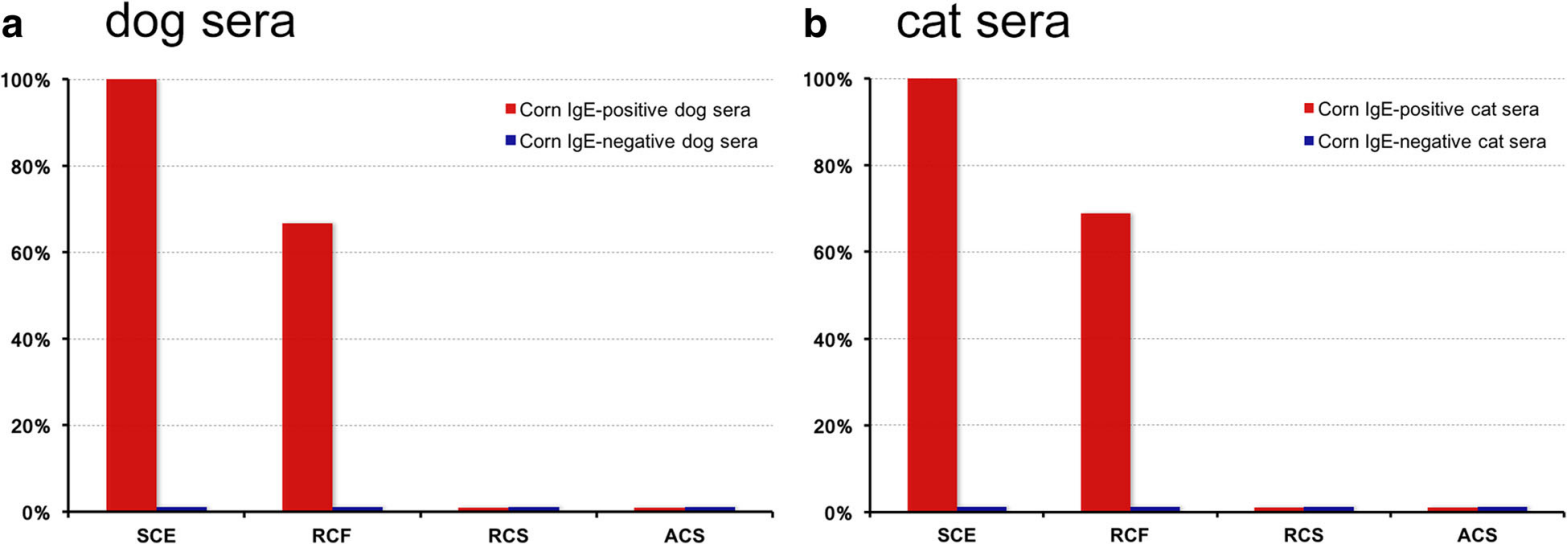
Frequencies of sera with positive reactions to the various extracts. **a** canine sera; **b** feline sera

The percentages of IgE reactivity of the four groups of canine sera to the four different extracts are shown in Table 1 and, aggregated, in Fig. 1a.

**Table 1:** Frequencies of canine and feline sera with positive reactions to the various extracts

Canine Sera	D1-LCoR	D1-MCoR	D1-HCoR	D2-NCoR
Standard corn extract	100%	100%	100%	0%
Regular corn flour extract	0%	100%	100%	0%
Regular corn starch extract	0%	0%	0%	0%
Anallergenic corn starch extract	0%	0%	0%	0%
Feline Sera	C1-LCoR	C1-MCoR	C1-HCoR	C2-NCoR
Standard corn extract	100%	100%	100%	0%
Regular corn flour extract	22%	80%	100%	0%
Regular corn starch extract	0%	0%	0%	0%
Anallergenic corn starch extract	0%	0%	0%	0%

All sera from the D1 group (i.e., corn-reactive sera) had IgE against the SCE. Among these sera, all those with moderate and high corn-specific IgE levels (i.e. 20/30 sera, 67%) also had IgE identifying allergens in the flour (RCF). Importantly, none of the forty canine sera had positive levels of IgE against allergens present in the two cornstarch extracts (RCS and ACS).

Similarly and as expected, none of the sera from the D2 group (corn-nonreactive sera) had measurable IgE against any of the four tested extracts.

### Feline ELISAs

The percentage of reactivity of the feline sera to the various extracts are also compiled in Table 1 and Fig. 1b.

Altogether, all 29 feline corn-reactive sera (C1-LCoR, MCoR, and HcoR) had positive levels of IgE that recognized SCE proteins, 20/29 (69%) contained IgE-specific for allergens in the flour while none had measurable IgE against starch allergens.

Sera from corn-nonreactive cats (C2-NCoR) did not have any positive IgE reactivity to any of the tested extracts.

### Immunoblotting

We used SDS-PAGE to separate the proteins according to their molecular weight under reducing and denaturing conditions. Multiple bands in the range of approximately 97–10 kDa were visible in the SCE lane (Additional file 1), with distinct bands at approximately 97, 90, 77, 52, 42, and 38 kDa. Faint bands of molecular weight of approximately 21, 10, 6, and 4 kDa were visible in the lane with the RCF extract (Additional file 1). There were no bands visible in lanes separating proteins from both starches (Additional file 1, RCS and ACS lanes). Importantly, both SCE and RCF extracts had a low molecular weight band of 9–10 kDa, which was not detected in both starches.

**Fig. 2:**
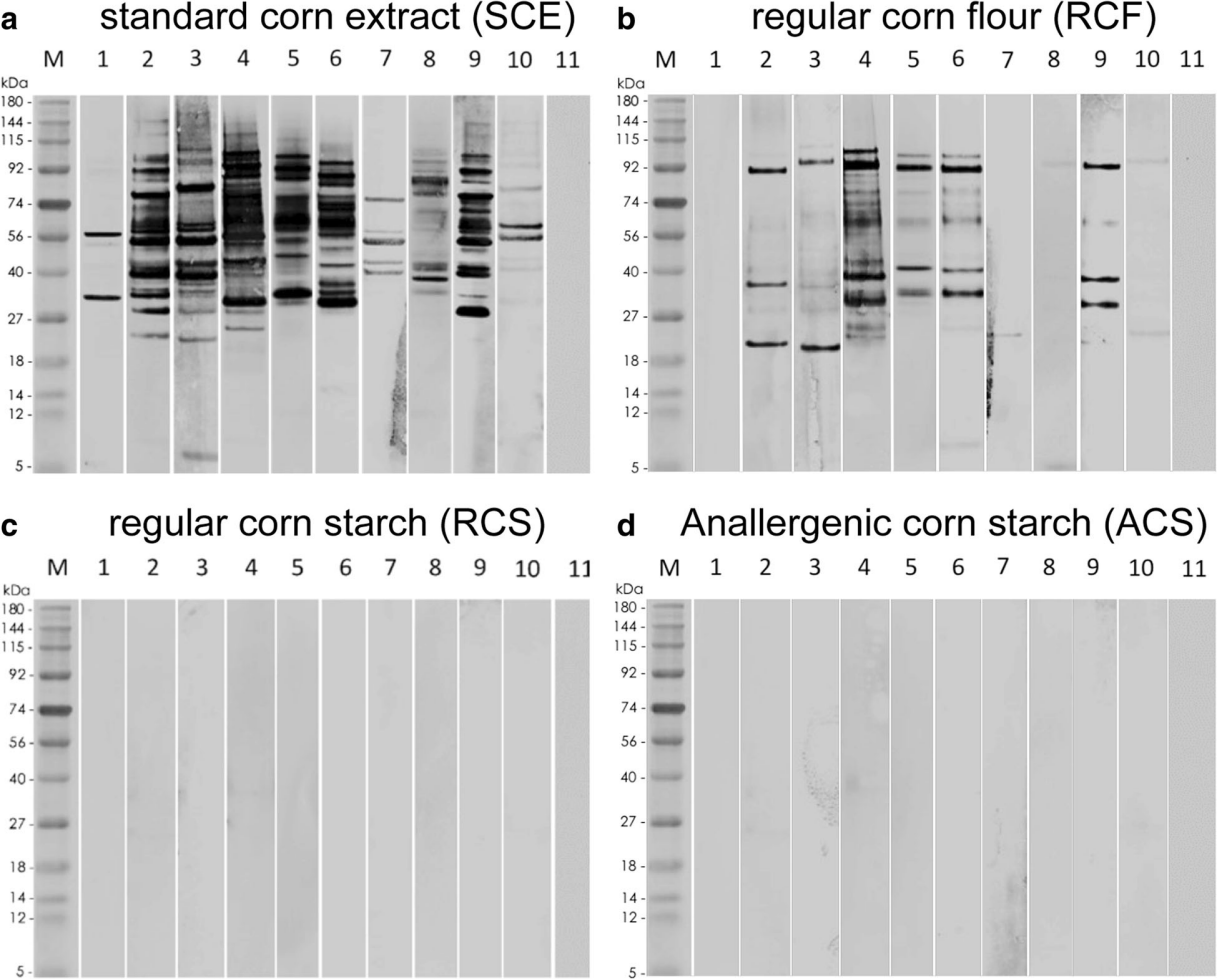
Immunoblotting performed with canine sera. **a** SCE; **b** RCF; **c** RCS; **d** ACS. Lane M: molecular weight (kDa) markers; lanes 1–10: corn-sensitized dog sera 1–10, respectively; lane 11: corn-negative dog pool

Using immunoblotting, we observed that IgE from the ten corn-sensitized dogs recognized proteins of molecular weight between approximately 8 and 141 kDa in the SCE, with all dogs sensitized to at least four proteins (Fig. 2a). Major allergens, that is those recognized by 50% or more of tested corn-reactive sera were of approximately 101 (6/10 dogs; 60%), 92 (90%), 87 (70%), 80 (70%), 72 (60%), 61 (70%), 41 (90%) and 29 kDa (70%) (Fig. 2a).

Nine of ten corn-sensitized dogs (90%) had detectable IgE that recognized RCF proteins of molecular weight between 4 and 103 kDa; major corn flour allergens were those of 93 (8/10 dogs; 80%), 61 (50%), 37 (70%), 30 (60%) and 22 kDa (70%) (Fig. 2b). The binding of IgE to proteins in the SCE and RCF was variable, in both intensity and number, between individual dogs (Fig. 2a, b); common major allergens in the corn kernel and flour extracts were those of 92/93, 29/30 and 61 kDa.

In contrast to the results above, there was no binding of SCE-positive dog serum IgE to RCS or ACS extracts (Fig. 2c, d). Similarly, there was no IgE reactivity in the SCE-negative dog serum pool control blots (Fig. 2a-d).

**Fig. 3:**
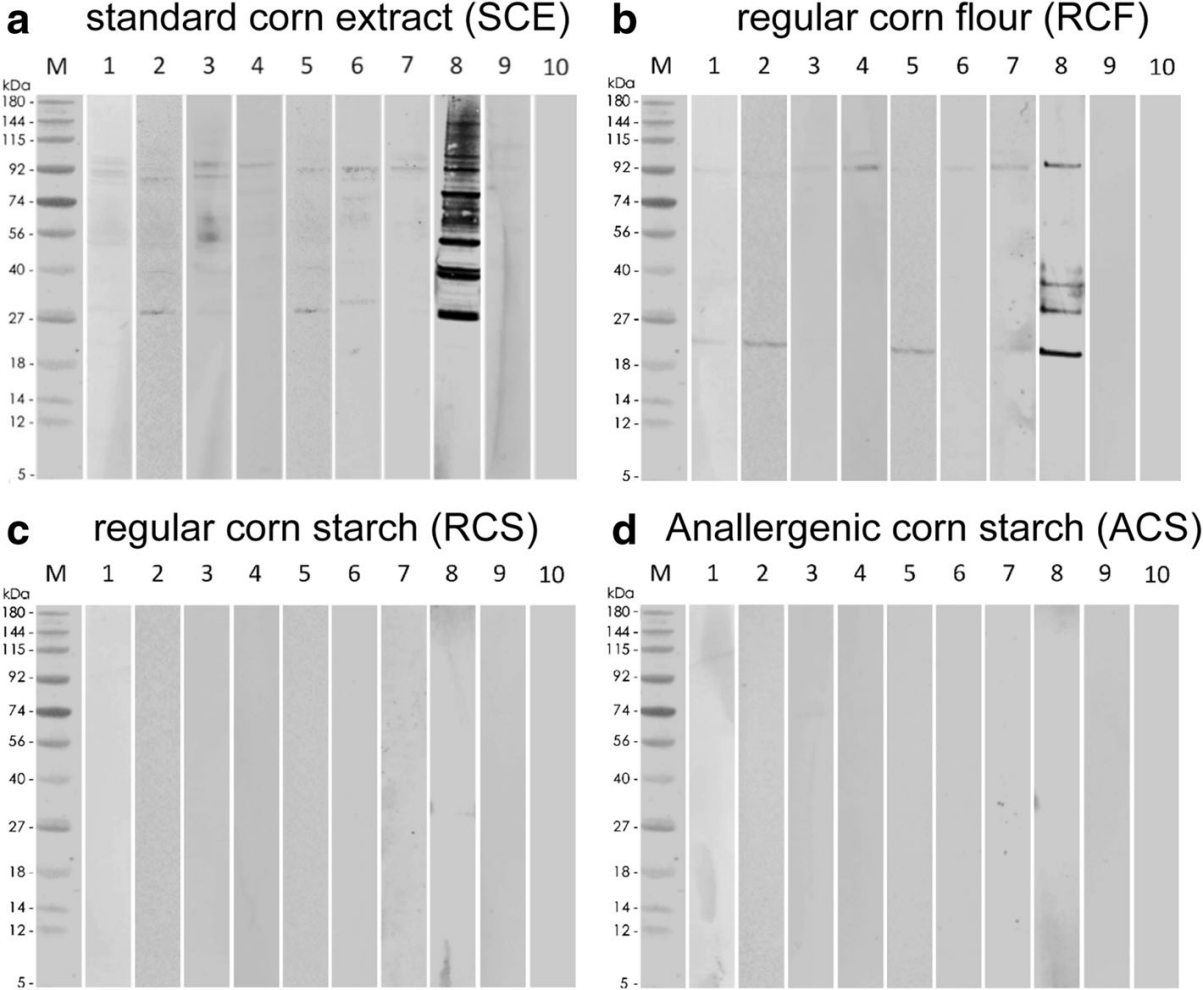
Immunoblotting performed with feline sera. **a** SCE; **b** RCF; **c**: RCS; **d** ACS. Lane M: molecular weight (kDa)markers; lanes 1–9: corn-sensitized cat sera 1–9, respectively; lane 10: corn-negative cat serum pool

The results from corn-sensitized feline serum IgE binding to proteins of SCE, RCF, RCS and ACS are shown in Fig. 3a-d, respectively. Cat IgE reacted to SCE proteins of molecular weight between approximately 28 and 150 kDa with all sera binding to at least one SCE protein. Major corn allergens were those of 100 (5/9 cats; 56%), 93 (7/9; 78%), 87 (6/9; 67%), 56 (67%), 41 (67%) and 29 kDa (78%) (Fig. 3a).

In the flour, feline serum IgE bound to proteins of molecular weight between 20 and 106 kDa, with all but one cat having a detectable reactivity to the RCF (Fig. 3b). Major corn flour allergens for corn-sensitized cats were proteins of approximately 93 (8/9 cats; 89%) and 21 kDa (5/9 cats; 56%) (Fig. 3b). Similarly to dogs, IgE responses in individual cats to allergens present in the SCE and RCF extracts were highly variable with the 93 kDa protein being a common major allergen.

In contrary to the results above and similarly to dogs, there was no binding of SCE-positive cat serum IgE to RCS or ACS extracts and no IgE reactivity in SCE-negative cat serum pool control blots.

## Discussion

In this serological study using two different techniques, IgE detected allergens in corn flour only in dogs and cats having moderate and high IgE serum levels against a corn kernel extract. In contrast, none of the tested sera had detectable IgE against cornstarch proteins. As such, cornstarch should be considered hypoallergenic compared to its flour in dogs and cats with detectable serum corn-specific IgE.

These results are not surprising, as they echo the clinical indings of experimental challenges in corn-sensitized dogs [11]. In this study, 14 Maltese-beagle atopic dogs with historical clinical hypersensitivity to corn and soy were challenged, in three separate phases of two consecutive administration days, with 200 mg/kg of cornmeal (i.e., kernels ground to a coarser texture than flour), cornstarch and soy [11]. While ten of these 14 dogs (71%) exhibited a flare of clinical signs after eating the cornmeal, only three (21%) flared with cornstarch, these three dogs also having reacted to cornmeal. Consequently, only three of ten dogs (30%) clinically-reactive to cornmeal had a flare after eating the cornstarch [11].

Even though cornstarch contains approximately less than one-twentieth of the protein amount of corn flour (0.3 vs 6.9 g of protein/100 g; http://nutritiondata.self.com; page last accessed October 31, 2017), the lower allergenicity that was seen with the starch is unlikely due to a difference in protein concentration between these foodstuffs. Indeed, we performed ELISAs and immunobloting with the same protein concentration (5 μg/ml) for all extracts. Consequently, the difference in the allergenicity of corn byproducts most likely resides in the different protein composition existing between flour and starch.

While corn flour is made from milling whole dried kernels, cornstarch mostly contains the kernel’s starch granule-rich endosperm. In addition to amylose and amylopectin, seed endosperms also include storage proteins for the embryo as well as structural, metabolic and starch granule-associated proteins (SGAPs) [12].

Recent proteomic studies employed 2D-electrophoresis and mass spectrometry to characterize corn kernel allergens recognized by IgE from corn-allergic human sera [5–7]. Altogether, more than 20 allergens were uncovered in corn flour [5–7]. At the time of this writing, the allergen standardization international registry (www.allergen.org; site last accessed October 31, 2017) only lists three of these corn kernel allergens (Zea m 8, 14 and 25).

Matching the recently identified corn kernel allergens [5–7] to proteins characterized in a cornstarch proteome confirms the relative hypoallergenicity of cornstarch compared to its parent flour [13]. Indeed, the starch appears to contain only half of the newly-recognized corn kernel allergens [13]. Five of these are enzymes involved in carbohydrate metabolism: the GBSS-I (NDP-glucose-starch glucosyltransferase), the UTP-glucose-1-phosphate uridylyltransferase, the cytoplasmic isozyme of fructose-bisphosphate aldolase, the cytosolic triosephosphate isomerase and the malate dehydrogenase [5–7, 13]. Other cornstarch allergens are seed storage proteins: the globulin 2 (Zea m G2), the vicilin-like embryo storage protein (Zea m G1) and several proteins of the zein and glutelin families (including Zea m 27). Finally, the starch allergen thioredoxin H2 (Zea m 25) is involved in protein folding, sorting and degradation [13]. Importantly, cornstarch does not appear to contain the major corn kernel allergen, the 9 kDa lipid-transfer protein Zea m 14 [8], a finding that we confirmed with the likely detection of this allergen with its typical 9–10 kDa band in the flour but not in the two tested starches.

While the specific amounts of each of these allergenic proteins were not reported in the two cornstarch proteomic articles [13, 14], one can infer from the published data that over half of the SGAPs are enzymes involved in carbohydrate metabolism [13]. In the earlier study, the protein(s) present in largest amount in cornstarch were the granule-bound starch synthase(s) (GBSSs) [14]. The granule-bound starch synthase family was later confirmed to compose up to 85% of the total internal SGAPs [13]. Similarly, mass spectrometry analysis of the Royal Canin Anallergenic itself, which contains hydrolyzed poultry feathers and the purified cornstarch tested herein, identified only residual GBSS-I [15]. It is likely that, during the desiccation of the endosperm for starch processing, these normally cytoplasmic amylogenesis enzymes remained bound to the remnants of the amyloplast membrane of the starch granules [16].

Importantly, the GBSS-I was reported recently to be an allergen for both corn-sensitized humans [7, 17] and dogs [9]. Unfortunately, because of the high homology existing between GBSSs from starch-containing plants, that the GBSS-I is an allergen leads to a high risk of IgE cross-reactivity, not only among grains [18], but also likely between grains and tubers [9, 19]. Whether such cross-reactivity would be clinically-relevant in corn-allergic humans or dogs has not been reported yet.

That proteins like GBSS-I are recognized corn allergens does not mean that they could induce flares in corn-allergic individuals, be it an animal or a human. There are at least three factors that could reduce its allergenicity: 1) the nature of the targeted epitopes, 2) food processing and 3) the amount of allergen consumed.

While most allergic patients have IgE that recognize epitopes on the peptidic moiety of proteins, others bind to glycans on complex glycoproteins (reviewed in [20]). The most common situation is when IgE targets classic xylose-containing cross-reactive carbohydrate determinants (CCDs) that are shared among proteins of a plant, insect and parasite origin [20]. Identifying that IgE target classic CCDs is essential, as they could be the source of nonspecific IgE seropositivity as such IgE are believed to be mostly nonpathogenic in humans [20]. To address this issue, we previously evaluated if the reactivity of corn-specific IgE in dogs was specific for glycans. Sera from seven dogs with high corn-specific IgE were tested against the same SCE as above, the CCD-rich bromelain (a protein from pineapple) and a high-amylose cornstarch different from those examined in the current study; testing was done by ELISA before and after deglycosylation of the extracts using a standard periodate oxidation protocol. While the median post-deglycosylation reduction of IgE reactivity to the SCE was only about 20%, that to bromelain was close to 50%, and that to the high-amylose cornstarch was above 80% (J. Bexley: unpublished data). Results of this pilot study suggest that, even if present, most of the IgE reactivity to cornstarch proteins appears directed against CCDs, and, as such, might not be pathogenic or relevant.

Food processing is another reason why allergens would no longer be recognized by IgE from clinically-reactive patients, for example, if allergens were thermolabile. This phenomenon has been shown recently to also occur in dogs suspected of having a food allergy: raw proteins seemed more allergenic than cooked ones [21, 22]. Conversely, it is possible, at least theoretically, that the detection of the GBSS-I by IgE in the study by Roitel and colleagues [9] might be due to allergenic neo-epitopes being induced by heat and processing during pet food manufacturing, while, in our study, we used corn starches before their inclusion and processing in the diet itself.

Furthermore, the clinical reactivity of food-allergic humans is mainly allergen-dose-dependent, with protein doses eliciting reactions varying markedly between children and adults or between food sources [23]. For example, while less than 0.1 mg of milk proteins might be sufficient for some milk-allergic children to flare, adults might need 50 times that amount; for shrimp, more than 10 mg of protein would be required for 5% of adults to react [23]. How much GBSS-I would be eaten by a dog fed a cornstarch-containing pet food and whether its allergenicity would survive after food processing is unknown. Nevertheless, our immunoblotting studies showed that, while some corn-sensitized dogs had IgE directed against SCE and SCF proteins of a molecular weight compatible with that of the GBSS-I (55–60 kDa), such bands were remarkably absent with the two tested starch extracts. These results suggest that, even if detectable by mass spectrometry in the Royal Canin Anallergenic [15], the amount of residual GBSS-I present is likely minute. It is only by challenging corn-allergic dogs with cornstarch processed in pet foods that the real determination of the allergenicity of GBSS-I will be thoroughly assessed.

Finally, the immunoblotting experiments provided valuable data on the molecular weights of several major corn allergens recognized by corn-sensitized dogs and cats. It is hoped that the sequencing of these bands will help precisely determine the identity of such allergens and their respective amount in corn-derived products such as flour and starch.

## Conclusions

In this study, we established, using both ELISA and immunoblotting, that IgE from corn-sensitized dogs is less likely to recognize cornstarch proteins than those present in kernel and flour extracts. As corn is not yet known as a common allergen source in dogs and cats, and as its starch seems to be less allergenic than the flour, pet foods containing cornstarch as a carbohydrate source might be preferable for pets suspected of corn allergies.

## Additional file

Additional file 1 : SDS-PAGE. Extracts (~ 5 μg/lane) were separated in 4–12% gels by SDS-PAGE. Lane 1: molecular weight (M wt) markers; lane 2: standard corn extract (SCE); lane 3: regular corn flour (RCF) extract; lane 4: Regular cornstarch (RCS) extract; lane 5: Anallergenic cornstarch (ACS) extract. (TIFF 218 kb)

## Abbreviations

ACS: Anallergenic cornstarch (Royal Canin)
CAFR: Cutaneous adverse food reaction
HCoR: High corn-reactive sera
LCoR: Low corn-reactive sera
MCoR: Medium corn-reactive sera
NCoR: Corn non-reactive sera
OD: Optical density
RCF: Regular corn flour
RCS: Regular cornstarch
SCE: Standard corn extract
