# Supplementary figures and images for: Cornstarch is less allergenic than corn flour in dogs and cats previously sensitized to corn
Source: BMC Vet Res. 2018 Jun 27;14:207. doi: 10.1186/s12917-018-1538-5 (PMC6020376; doi:10.1186/s12917-018-1538-5)

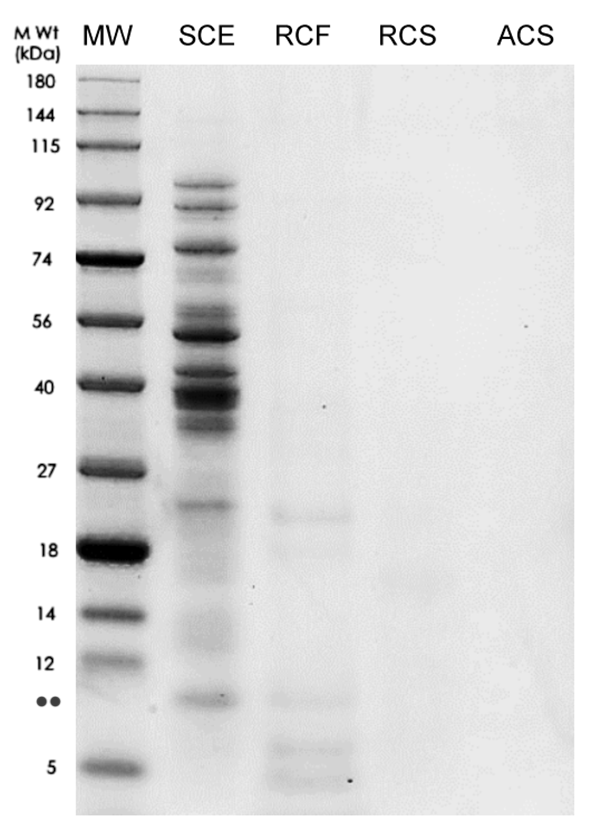

Supplement: Supplementary file 1 — : SDS-PAGE. Extracts (~ 5 μg/lane) were separated in 4–12% gels by SDS-PAGE. Lane 1: molecular weight (M wt) markers; lane 2: standard corn extract (SCE); lane 3: regular corn flour (RCF) extract; lane 4: Regular cornstarch (RCS) extract; lane 5: Anallergenic cornstarch (ACS) extract. (TIFF 218 kb) [file 12917_2018_1538_MOESM1_ESM.tiff]
